# HAUSP-nucleolin interaction is regulated by p53-Mdm2 complex in response to DNA damage response

**DOI:** 10.1038/srep12793

**Published:** 2015-08-04

**Authors:** Key-Hwan Lim, Jang-Joon Park, Bon-Hee Gu, Jin-Ock Kim, Sang Gyu Park, Kwang-Hyun Baek

**Affiliations:** 1 Department of Biomedical Science, CHA University, Bundang CHA General Hospital, Gyeonggi-Do 463-400, Republic of Korea.; 2 College of Pharmacy, Ajou University, Suwon, Gyeonggi-Do 443-749, Republic of Korea

## Abstract

HAUSP (herpes virus-associated ubiquitin specific protease, known as ubiquitin specific protease 7), one of DUBs, regulates the dynamics of the p53 and Mdm2 network in response to DNA damage by deubiquitinating both p53 and its E3 ubiquitin ligase, Mdm2. Its concerted action increases the level of functional p53 by preventing proteasome-dependent degradation of p53. However, the protein substrates that are targeted by HAUSP to mediate DNA damage responses in the context of the HAUSP-p53-Mdm2 complex are not fully identified. Here, we identified nucleolin as a new substrate for HAUSP by proteomic analysis. Nucleolin has two HAUSP binding sites in its N- and C-terminal regions, and the mutation of HAUSP interacting peptides on nucleolin disrupts their interaction and it leads to the increased level of nucleolin ubiquitination. In addition, HAUSP regulates the stability of nucleolin by removing ubiquitin from nucleolin. Nucleolin exists as a component of the HAUSP-p53-Mdm2 complex, and both Mdm2 and p53 are required for the interaction between HAUSP and nucleolin. Importantly, the irradiation increases the HAUSP-nucleolin interaction, leading to nucleolin stabilization significantly. Taken together, this study reveals a new component of the HAUSP-p53-Mdm2 complex that governs dynamic cellular responses to DNA damage.

Posttranslational modification of numerous proteins in eukaryotic cells relies on the counterbalancing effect of ubiquitination and deubiquitination. Most proteins contain at least one or more lysine specific ubiquitination sites, and the ubiquitination process is catalyzed by the sequential actions of E1 ubiquitin-activating, E2 ubiquitin-conjugating, and E3 ubiquitin ligase enzymes, followed by protein transfer to the 26S proteasome. This process is referred as the ubiquitin proteasome pathway (UPP)^1^. In addition to the monoubiquitin chain, free ubiquitins can be conjugated to ubiquitin molecules attached to target proteins to link polyubiquitin chains. Structural and functional analyses of polyubiquitin chains indicate that polyubiquitin chains can make diverse conformation depending on ubiquitination of its lysine residues at Lys6, Lys11, Lys27, Lys29, Lys33, Lys48 or Lys63, and these are involved in the regulation of intracellular signaling^2^. The ubiquitination process is reversible and mono- or poly-ubiquitin chains can be removed by various deubiquitinating enzyme (DUBs)^3^. Approximately, ~100 DUBs are encoded in human genome that can be classified into at least six families; ubiquitin-specific proteases (USPs), ubiquitin C-terminal hydrolases (UCHs), ovarian tumor proteases (OTUs), Machado-Josephin domains (MJDs), JAB1/MPN/MOV34 (JAMMs), and monocyte chemotactic protein-induced proteases (MCPIPs)^4^. The USP, UCH, OTU and MJD are known as cysteine proteases, and JAMM is known as metalloproteases^5^,^6^. The USPs specifically detach covalently bound ubiquitins from lysine sites to regulate substrate stabilization and intracellular localization^7^. Recent studies have shown that the USP family contains three major catalytic functional domains of Cys, His, and Asp boxes. These conserved domains of USPs are responsible for cleaving the monoubiquitin or polyubiquitin chains^8^.

HAUSP (herpes virus-associated ubiquitin specific protease; also known as USP7), a cysteine isopeptidase of the USP family, is known to regulate cell growth and apoptosis associated with stabilization of p53 from ubiquitin-mediated degradation^9^,^10^. HAUSP knockout mice have been generated to identify *in vivo* function of HAUSP, but the mice showed embryonic lethality between embryonic day E6.5 and E7.5, indicating that HAUSP is an important protein for mouse development^11^. The functions of HAUSP in cellular regulation have been shown that it is involved in oxidative stressed response^12^ and epigenetic modification^13^,^14^,^15^. Recently, TSPYL5 as an inhibitory protein for HAUSP was identified repressing the expression of *HAUSP* gene^16^. Several studies have demonstrated that HAUSP also deubiquitinates and elongates the half-life of Mdm2 as well as p53, and that these three proteins can form a complex^17^,^18^. Both p53 and Mdm2 have been proposed to contain a P/AXXS motif in their C-terminal domains, and these amino sequences mediate the interaction with HAUSP^19^. HAUSP is phosphorylated on Ser18 by CK2 protein kinase, and this phosphorylation leads to the inhibition of Mdm2 ubiquitination^20^. However, upon DNA damage, ATM-dependent activation of PPM1G protein phosphatase leads to HAUSP dephosphorylation. Dephosphorylated HAUSP undergoes the proteasomal degradation, and this causes decrease of Mdm2 stability, leading to an increase in the p53 level^20^. Thus, HAUSP is postulated to play pivotal roles in p53-Mdm2 protein regulation in the DNA damage response, but the precise molecular mechanism governing its regulation is not completely understood. In a previous study, we performed a two dimensional electrophoresis (2-DE) and MALDI-TOF/MS analysis, using HAUSP-overexpressing HeLa cells to isolate new proteins operating in the HAUSP-p53-Mdm2 network^21^. We found novel HAUSP binding proteins including nucleolin as an rRNA synthesis regulating protein and demonstrated that the HAUSP-nucleolin interaction is involved in regulating the p53-Mdm2 complex in response to DNA damage.

## Results

### Nucleolin is associated with HAUSP

**Figure 1:**
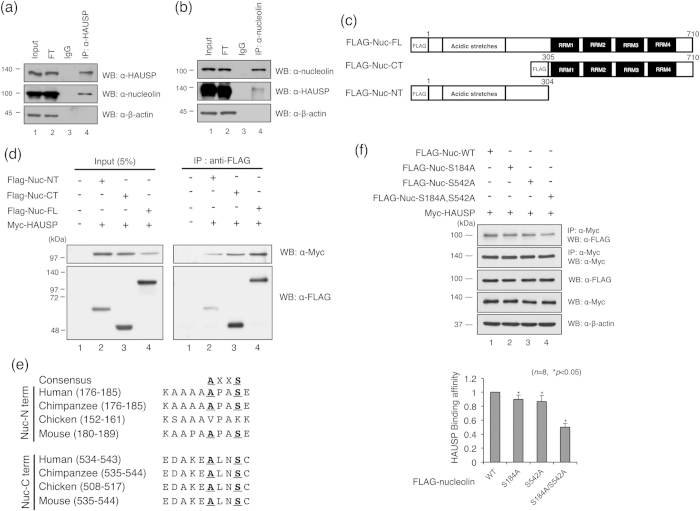
Nucleolin associates with HAUSP via its HAUSP binding motif. (**a**) HeLa cell lysates were precipitated by an anti-HAUSP antibody. HAUSP and nucleolin were detected by anti-HAUSP and anti-nucleolin antibodies, respectively. (**b**) HeLa cell lysates were precipitated by an anti-nucleolin antibody. HAUSP and nucleolin were detected by appropriate antibodies. The β-actin was used as a loading control for Input and FT (FT, flow-through; IP, immunoprecipitate). (**c**) the peptide map of wild-type nucleolin, N-terminal and C-terminal deletion mutants. (**d**) HEK 293T cells were transfected with a Myc-tagged HAUSP, FLAG-tagged N- and C-terminal deletion mutants of nucleolin. Lysates from these cells were incubated with an anti-FLAG antibody, and a protein complex was immunoblotted with an anti-Myc antibody. (**e**) mapping of the HAUSP binding site of nucleolin in mammalian species. (**f**) lysates from HEK 293T cells transfected with Myc-tagged HAUSP and FLAG-tagged nucleolin (S184A, S542A and S184/S542A) were immunoprecipitated with an anti-Myc antibody, and Western blotting was performed with indicated antibodies. Statistical data are presented as a means (*n* = 8, *p < 0.05).

Previously, we have screened HAUSP interacting proteins by proteomic analysis with HAUSP-overexpressing HeLa cells and investigated the interaction between HAUSP and HAUSP binding proteins^21^. Among these candidates, nucleolin is particularly intriguing as it is known to associate with p53 in the nucleus^22^. Moreover, nucleolin inhibits E3 ubiquitin ligase activity of Mdm2 thereby causing the elevation of p53 level^23^. Regulation of Mdm2 and p53 by HAUSP is mediated by a complex formation^18^. Consistent with previous studies, it is presumed that nucleolin could be involved in HAUSP-Mdm2-p53-mediated cell stress and DNA damage responses. Therefore, we next evaluated the interaction between HAUSP and nucleolin (Fig. 1a,b). Nucleolin was detected by coimmunoprecipitation using an anti-HAUSP antibody, and reciprocal immunoprecipitation with an anti-nucleolin antibody also brought down HAUSP (Fig. 1a,b). We determined the specific interaction domains between HAUSP and nucleolin by generating FLAG-tagged N-terminal (AA1–304) and C-terminal (AA305–710) regions of nucleolin (Fig. 1c). Mapping the region of nucleolin showed that both N- and C-terminal regions of nucleolin interacted with HAUSP (Fig. 1d). The N-terminal region of nucleolin seemed to have a higher molecular weight when compared to the C-terminal region. This is because the N-terminal domain of nucleolin has acidic stretches, and this may affect protein mobility on the gel^24^,^25^. Furthermore, the full-length of nucleolin binds more efficiently with HAUSP compared to N- and C-terminal regions of nucleolin (Fig. 1d).

The amino acid sequence P/AXXS is well known to act as a binding motif for HAUSP^19^; therefore, we reasoned that the ability of nucleolin to bind HAUSP is due to its AXXS sequences in both its N- and C- terminal regions (Fig. 1e). We tested this idea by generating mutants, in which the AXXS binding motifs of the N- and C-terminals of nucleolin were both changed from Ser to Ala (Nuc-S184A, Nuc-S542A, and Nuc-S184A/Nuc-S542A). We then performed immunoprecipitation using three nucleolin mutant constructs. As shown in Fig. 1f, the interaction of nucleolin mutants with HAUSP was decreased when compared to the wild-type nucleolin. The interaction between the double mutant form of nucleolin (Nuc-S184A/S542A) and HAUSP was also markedly reduced. Together, these results demonstrate that nucleolin strongly binds to HAUSP through its N- and C-terminal regions, and that AXXS sequences in both the N- and C- terminal regions of nucleolin are critical for binding HAUSP.

**Figure 2:**
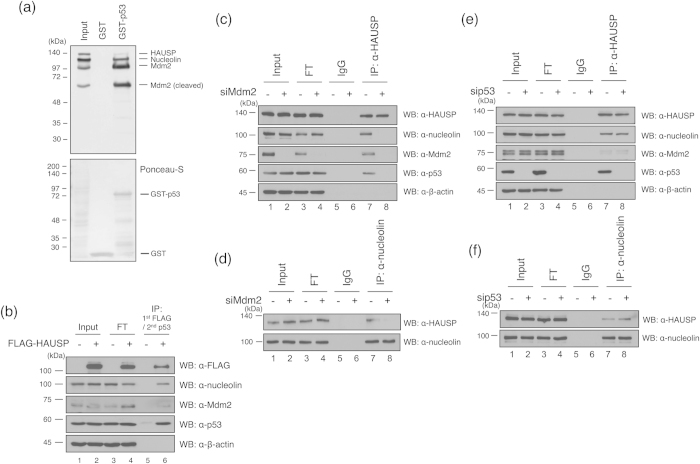
Nucleolin makes a complex with HAUSP, p53, and Mdm2. (**a**) HEK 293T cell lysates were incubated with GST-tagged p53. The complex of HAUSP, Mdm2, p53, and nucleolin was detected by respective antibodies (top). The bottom panel shows Ponceau-S staining of the membrane. (**b**) U2OS cells were transfected with FLAG-tagged HAUSP and the cell lysates were first immunoprecipitated with M2 FLAG-coated beads, and followed by the second immunoprecipitation performed with an anti p53 antibody. Western blotting was performed with anti-FLAG, anti-nucleolin, anti-Mdm2 and anti-p53 antibodies. (**c**,**d**) U2OS cells transfected with Mdm2, (**e**,**f**) p53 and control siRNA using RNAiMAX. After immunoprecipitation with anti-HAUSP or anti-nucleolin antibodies, Mdm2, p53, nucleolin, HAUSP and β-actin were detected using appropriate antibodies.

Next, we investigated whether nucleolin might be a component of the HAUSP-Mdm2-p53 complex. Previous studies had revealed that nucleolin binds Mdm2 to regulate p53 ubiquitination, and HAUSP can form a complex with Mdm2 and p53^18^,^23^. Therefore, we generated recombinant glutathione S-transferase (GST) tagged p53 (Fig. 2a, lower), and performed a GST pull-down assay using GST-p53. As expected, purified GST-p53 protein could bind Mdm2, nucleolin, and HAUSP (Fig. 2a, upper panel). In addition, we determined whether these proteins interact in a complex *in vivo*. To confirm this, we performed two-step serial immunoprecipitation assay using M2 FLAG-coated beads and an anti-p53 antibody with FLAG-tagged HAUSP overexpressing U2OS cells (Fig. 2b). As expected, the nucleolin was detected in HAUSP and p53 precipitates by Western blotting (Fig. 2b). We next verified that HAUSP and nucleolin could form a complex with p53 and Mdm2 by examining the interaction between HAUSP and nucleolin in p53- and Mdm2-depleted cells. Interestingly, reduction of Mdm2 by siRNA also significantly reduced the HAUSP and nucleolin binding (Fig. 2c,d). However, knockdown of p53 had no effect on the HAUSP-nucleolin interaction (Fig. 2e,f). Collectively, these results suggest that nucleolin is involved in the formation of the HAUSP-Mdm2-p53 complex, and the HAUSP-nucleolin interaction requires the presence of Mdm2 in normal condition.

### HAUSP regulates nucleolin stability via its deubiquitinating activity

**Figure 3:**
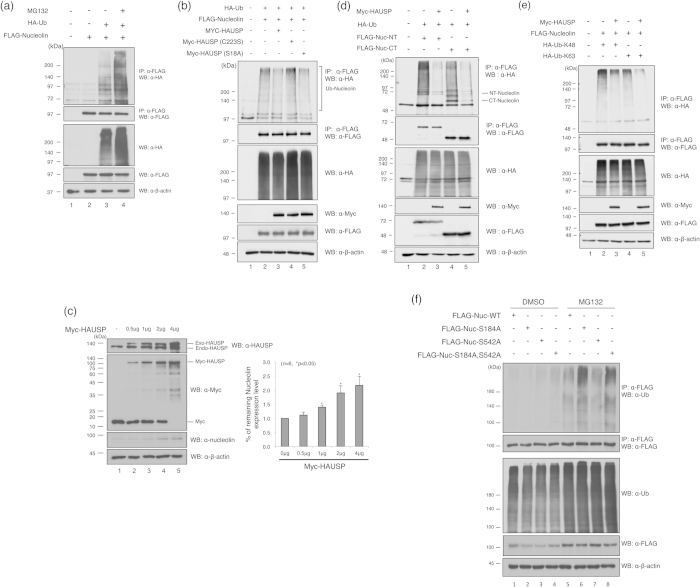
The nucleolin undergoes ubiquitination and is deubiquitinated by HAUSP. (**a**) HEK 293T cells expressing FLAG-tagged nucleolin and HA-tagged ubiquitin were treated with the proteasome inhibitor MG132 before harvest. Cell lysates were immunoprecipitated with an anti-FLAG antibody, and immunoblotted with an anti-HA antibody. (**b**) HEK 293T cell extracts with the overexpressed HA-tagged ubiquitin, FLAG-tagged nucleolin, Myc-tagged HAUSP and Myc-tagged HAUSP catalytic dead mutant (C223S) were immunoprecipitated with an anti-FLAG antibody, and ubiquitinated proteins were detected by an anti-HA antibody. The protein expression was analyzed by immunoblotting with a mixture of anti-FLAG and anti-Myc antibodies, and an anti-β-actin antibody was used as a loading control. (**c**) Myc-tagged HAUSP was transfected into HEK 293T cells by a dose-dependent manner as indicated, and the expression level of nucleolin was detected by an anti-nucleolin antibody. Statistical data are presented as a means (*n* = 6, *p < 0.05). (**d**) HEK 293T cells were transfected with HA-ubiquitin, the N- or C-terminal domain of FLAG-nucleolin, and/or Myc-HAUSP. After immunoprecipitation with an anti-FLAG antibody, ubiquitination level was detected by an anti-HA antibody. (**e**) HEK 293T cells were transfected with HA-ubiquitin (R48K) or HA-ubiquitin (R63K), FLAG-nucleolin and/or Myc-HAUSP. After immunoprecipitation with an anti-FLAG antibody, the ubiquitination level was detected by an anti-HA antibody. (**f**) HEK 293T cells were transfected with FLAG-tagged nucleolin (WT), nucleolin (S184A), or nucleolin (S542A), respectively. After transfection, the cells were treated with or without MG132 for 6 hrs before harvest. Immunoprecipitates were immunoblotted with an anti-ubiquitin or an anti-FLAG antibody. Whole cell lysates were also immunoblotted with an anti-ubiquitin, an anti-FLAG, and an anti-β-actin antibody.

We investigated the role of the interaction between HAUSP and nucleolin by testing whether HAUSP is a deubiquitinating enzyme for nucleolin. Previous studies had revealed that the treatment with the proteasome inhibitor MG132 results in accumulation of nucleolin in nucleolar foci^22^. Therefore, we tested whether nucleolin undergoes the ubiquitin-dependent proteasomal degradation. We observed polyubiquitinated nucleolin (Fig. 3a, lane 3), and the proteasome inhibitor MG132 substantially increased the polyubiquitination level of nucleolin (Fig. 3a, lane 4). The levels of Lys48- and Lys63-linked polyubiquitin chains on nucleolin were also increased in response to MG132 (Supplementary Figure S1). Next, we determined whether HAUSP affects nucleolin ubiquitination. Overexpression of wild-type HAUSP, but not a catalytic mutant (C223S) decreased nucleolin ubiquitination (Fig. 3b, lane 3). In addition to the deubiquitinating activity, the dose-dependent expression of HAUSP increased nucleolin protein levels (Fig. 3c). Since HAUSP interacts with both N- and C-terminal domains of nucleolin (Fig. 1d), we next investigated essential ubiquitination regions of nucleolin. Interestingly, both N- and C-terminal regions of nucleolin undergo ubiquitination, and HAUSP markedly reduced the ubiquitination levels of both regions (Fig. 3d).

Cells in the normal condition possess a protein kinase CK2 that phosphorylates HAUSP at Ser18; this leads to Mdm2 stabilization by activating HAUSP^20^. Therefore, we investigated whether a mutation on the phosphorylation site of HAUSP (S18A) might affect nucleolin ubiquitination, and determined that the phosphorylation mutant is also able to reduce the polyubiquitination of nucleolin (Fig. 3b, lane 5), indicating that deubiquitination of nucleolin may not depend on HAUSP phosphorylation in the normal condition. Polyubiquitin chains are assembled through the Lys48 and Lys63 residues of ubiquitin on target proteins, which are involved in several distinct signaling pathways^26^. In general, Lys48-linked polyubiquitination leads to the 26S proteasomal degradation, while Lys63-linked polyubiquitination mediates diverse cellular functions involving intracellular signaling^27^. However, recent studies showed that Lys63-linked polyubiquitination also functions in inducing proteasomal degradation^2^,^28^,^29^. Therefore, we examined the pattern of polyubiquitination and deubiquitination of nucleolin following HAUSP overexpression (Fig. 3e). We found that nucleolin can be conjugated with Lys48- and Lys63-polyubiquitin chains, and that HAUSP hydrolyzed both types of chains (Fig. 3e). Therefore, HAUSP not only prevents nucleolin degradation by the 26S proteasome but also regulates its functions. Next, we checked the ubiquitination of the three nucleolin mutants, and observed that the level of ubiquitinated nucleolin was significantly increased in the S184A and double mutation forms by MG132 treatment (Fig. 3f). These results indicate that nucleolin has multiple polyubiquitination sites and the stability of the nucleolin protein requires deubiquitinating activity of HAUSP, and the N-terminal region of nucleolin may associate with deubiquitination by HAUSP. Collectively, these results indicate that HAUSP is an essential deubiquitinating enzyme for nucleolin.

### HAUSP and nucleolin have a synergistic effect that modulates cancer cell proliferation

**Figure 4:**
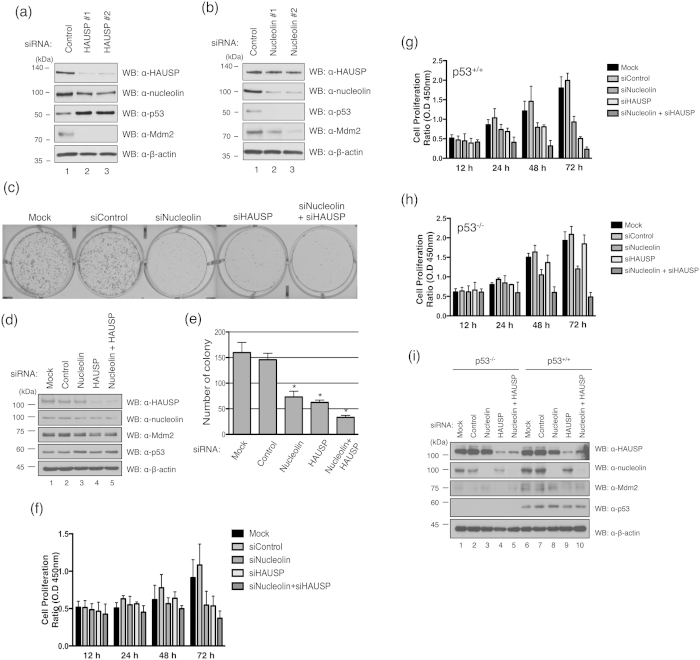
Knockdown effect of HAUSP and nucleolin in cancer cells. (**a**) U2OS cells were transfected with two different siRNAs for HAUSP knockdown, and the expression of HAUSP, nucleolin, p53, and Mdm2 was detected by indicated antibodies. (**b**) U2OS cells were transfected with two different siRNAs for nucleolin knockdown, and the expression level of proteins was detected by appropriate antibodies. (**c**,**d**) U2OS cells transfected with HAUSP and/or nucleolin siRNA were seeded onto 6-well plates. After 2 weeks, colonies were stained with crystal violet solution. (**d**) Expression of each protein was analyzed by Western blotting with indicated antibodies after transfection for 72 hrs. (**e**) The number of colonies represented in Figure c was counted. Significant difference from each sample was assessed by a Student’s *t*-test (n = 3, *p < 0.005). The number of colony was statistically analyzed in three separate experiments. (**f**) Cell proliferation assay. U2OS cells were transfected with nucleolin and/or HAUSP siRNA. At four different time points after post-transfection, cell proliferation ratio was analyzed using live cell staining buffer (CCK-8, cell counting kit-8), and then read using 450 nm filter. HCT116 p53 wild-type (+/+) **(g)** and knockout (−/−) (**h**) cells were transfected with nucleolin and/or HAUSP siRNA. At four different time points after post-transfection, cell proliferation ratio was analyzed using live cell staining buffer (CCK-8, cell counting kit-8), and then read using 450 nm filter. Significant difference from each sample was assessed by a Student’s *t*-test (n = 3, *p < 0.005). (**i**) Expression of each protein was analyzed by Western blotting with indicated antibodies after transfection for 72 hrs.

Tumor cell growth, volume, and weight were decreased by the depletion or inhibition of HAUSP^30^,^31^. Inactivation of nucleolin in cancer cells also led to a decrease in cell proliferation^32^. Accordingly, we next investigated the effect of HAUSP and nucleolin on cancer cell proliferation. Human osteosarcoma U2OS cells were transfected with siRNA against HAUSP or nucleolin (Fig. 4). HAUSP knockdown not only reduced HAUSP expression level but also decreased nucleolin level (Fig. 4a). The cells lacking HAUSP showed the increased level of p53 protein (Fig. 4a), consistent with a previous study^31^. Nucleolin knockdown also reduced the nucleolin expression, and it also affects the expression of p53 and Mdm2 (Fig. 4b)^23^. In addition, cell proliferation was reduced 2- to 3-fold by HAUSP or nucleolin depletion, respectively (Fig. 4c,d), and we observed a ~4 fold decrease in proliferation after HAUSP and nucleolin double-knockdown (Fig. 4c–e). To clarify the cell proliferation ratio in both nucleolin and HAUSP knockdown condition, we performed quantitative analysis with live cells that were transfected with nucleolin and/or HAUSP siRNA (Fig. 4f). The result showed that cell proliferation was inhibited by the knockdown of nucleolin, HAUSP, and nucleolin and HAUSP double-knockdown, respectively (Fig. 4f). Previous studies showed that both nucleolin and HAUSP regulate cell proliferation in a p53-dependent manner^9^,^23^, and we next verified the cell proliferation ratio in the absence or presence of p53 (Fig. 4g–i). The cell proliferation ratio of p53^−/−^ cells was higher than that in p53^+/+^ cells due to depletion of nucleolin, as expected (Fig. 4g,h). In addition, HAUSP, HAUSP and nucleolin double-knockdown cells also increase cell proliferation in p53^−/−^ cells compared to that in p53^+/+^ cells (Fig. 4g,h). These data support previous studies as HAUSP and nucleolin regulate cell proliferation in a p53-dependnet manner^9^,^23^.

### p53 and Mdm2 mediate a HAUSP-nucleolin interaction in response to DNA damage

**Figure 5:**
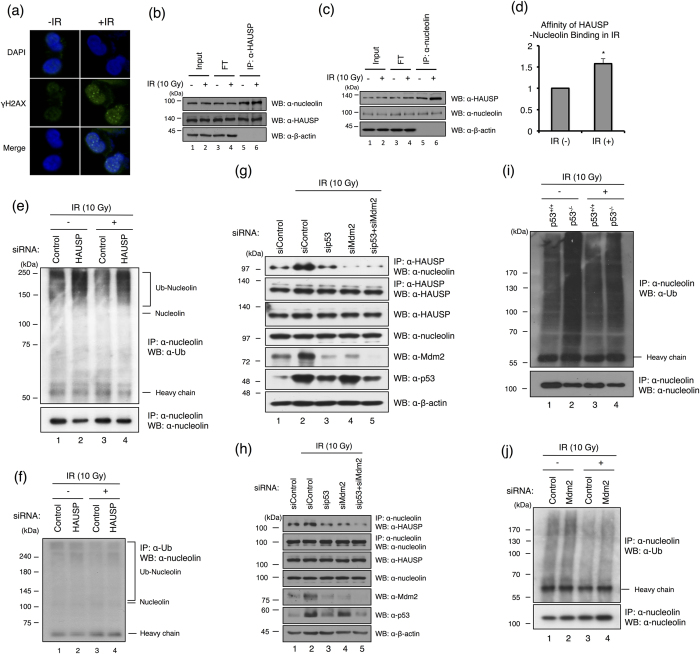
HAUSP controls the nucleolin stability via p53 and Mdm2 in DNA damage response. (**a**) U2OS cells were treated with IR (10 Gy), and processed immunofluorescence with an anti-γH2AX antibody (green) and DAPI (blue). (**b**,**c**) U2OS cell lysates exposed to IR (10 Gy) or controls were immunoprecipitated with anti-HAUSP or anti-nucleolin antibodies. Then, HAUSP and nucleolin were detected by appropriate antibodies. (**d**) Statistical data are presented as a means (n = 4, *p < 0.05). (**e**) U2OS cells transfected with HAUSP siRNA or control siRNA were exposed to IR (10 Gy). After immunoprecipitation with an anti-nucleolin antibody, the ubiquitination level of nucleolin was confirmed using an anti-ubiquitin antibody. (**f**) Reciprocal immunoprecipitation between nucleolin and ubiquitin with HAUSP siRNA transfected U2OS cell. (**g**,**h**) MCF7 cells transfected with p53, Mdm2, and control siRNA respectively, and cells were exposed to IR (10 Gy). HAUSP, nucleolin, Mdm2, and p53 were detected by appropriate antibodies after immunoprecipitation with an anti-HAUSP or an anti-nucleolin antibody. (**i**) HCT116 p53^+/+^ and p53^−/−^ cells exposed to IR (10 Gy) and cell lysates were immunoprecipitated with an anti-nucleolin antibody. Then, the ubiquitinated nucleolin was detected by an anti-ubiquitin antibody. (**j**) HCT116 cells transfected with control and Mdm2 siRNA, respectively, and cells were exposed to IR (10 Gy). Cell lysates were immunoprecipitated with an anti-nucleolin antibody. Then, the ubiquitinated nucleolin was detected by an anti-ubiquitin antibody.

Cell stress such as ionizing radiation (IR) regulates nucleolin mobilization from the nucleus to the nucleoplasm^33^. The interaction between nucleolin and p53 also increases after DNA damage, while the transcriptional level of nucleolin is not changed by p53^33^. Our findings that nucleolin is a novel substrate for HAUSP and is a component of the HAUSP-Mdm2-p53 complex led us to investigate the biological significance of HAUSP and nucleolin interaction in response to DNA damage. Therefore, we next examined the HAUSP and nucleolin interaction pattern in both normal and DNA damaged conditions. To evaluate DNA damage on cells, we checked the formation of the γH2AX foci as a marker for DNA double strand breaks with IR-treated U2OS cells (Fig. 5a). We next identified the interaction between HAUSP and nucleolin with IR-treated cells, and the result showed that the HAUSP and nucleolin interaction was increased in IR-treated cells (Fig. 5b–d), but no change was observed for HAUSP and nucleolin interaction in UV-treated cells (Supplementary Figure S2). A recent report showed that RNA polymerase I transcription is shut down in response to double strand breakdown (DSB)^34^, and that nucleolin represses RNA polymerase I transcription^35^. Therefore, we envisioned that nucleolin may be stabilized by HAUSP during the DNA damage. We next examined the stability of nucleolin during IR-induced damage. The ubiquitination level of nucleolin following knockdown of endogenous HAUSP was increased, as expected (Fig. 5e, lane 2). Interestingly, the ubiquitination level of nucleolin was reduced in the IR-treated cells, which may be associated with stabilization of nucleolin in response to IR-induced damage (Fig. 5e, lane 3). In addition, interference with HAUSP expression led to increase in the nucleolin ubiquitination level both in normal condition and with IR treatment (Fig. 5e, lanes 2 and 4), and a reciprocal interaction assay also showed the same result as shown in Fig. 5e (Fig. 5f). In the normal state, Mdm2-depleted cells, but not p53-depleted cells, reduced the binding affinity between HAUSP and nucleolin (Fig. 2). A previous study suggested that the interaction between HAUSP and Mdm2 is significantly decreased in response to DNA damage^36^. We hypothesized that Mdm2 acts as a mediator between HAUSP and nucleolin in the normal state, and that p53 may be associated with the HAUSP and nucleolin interaction in the DNA damaged condition. We tested this idea by treating cells with IR, where Mdm2 and/or p53 protein expression was suppressed by siRNA. As expected, the HAUSP-nucleolin interaction was reduced by interference with p53 expression by siRNA (Fig. 5g). Moreover, both Mdm2 alone and Mdm2/p53 double knockdown led to a dramatic reduction in the HAUSP-nucleolin interaction in IR treatment condition (Fig. 5g). A reciprocal interaction assay also showed the same result as shown in Fig. 5g (Fig. 5h). Next, we investigated whether the nucleolin stabilization by HAUSP depends on p53 upon DNA damage. The ubiquitination of nucleolin is increased by the absence of p53 (Fig. 5i), and showed the same with the HAUSP depletion (Fig. 5e,f). However, inhibition of the Mdm2 expression by Mdm2 siRNA did not affect to the ubiquitination of nucleolin in both normal and IR-treated condition (Fig. 5j). Taken together, our results demonstrate that the HAUSP and nucleolin interaction is mediated by p53 and Mdm2, and that these two proteins might act as mediators for the interaction between HAUSP and nucleolin.

## Discussion

The deubiquitinating enzyme HAUSP plays a major regulatory role in the p53-mediated DNA damage response pathway. Structural analysis of the HAUSP binding motif in Mdm2 and p53 confirmed a HAUSP-p53-Mdm2 interaction^19^. Crystal structure analysis visualized HAUSP-p53 and HAUSP-Mdm2 interactions via HAUSP binding motif P/AXXS^19^. The HAUSP binding motif P/AXXS was also confirmed by Epstein-Barr nuclear antigen-1 (EBNA1) protein interaction study^37^. The study showed that EBNA1 also has these peptide residues found in p53 and Mdm2^19^. More recently, it was demonstrated that UbE2E1 has the P/AXXS sequence in its N-terminal domain and HAUSP interacts with UbE2E1 via this conserved motif 37. Previously, we have screened HAUSP binding proteins and identified as Annexin a1 (known as ANXA1), PKM2, PP2A, and nucleolin^21^. We also identified the role of HAUSP upon UV damaged condition through the regulation of ANXA1^21^. In this study, we investigated the function of HAUSP with one of HAUSP interacting proteins, nucleolin, upon IR damaged condition. Nucleolin is subjected to the proteasomal degradation via ubiquitination, and is stabilized by HAUSP. Notably, the binding ability of both the N- and C- terminal regions of nucleolin to HAUSP could be explained by the existence of the HAUSP binding motif P/AXXS in both of these regions in the nucleolin (Fig. 1e,f).

The HAUSP-p53 and HAUSP-Mdm2 complexes have previously been characterized in the DNA damage response^17^,^18^. Following dephosphorylation by PPM1G in response to DNA damage, dephosphorylated HAUSP undergoes the proteasomal degradation through the ATM kinase pathway, thereby leading to downregulation of Mdm2 and upregulation of p53 protein levels^20^. However, the linkage between the DNA damage response machinery and the HAUSP-p53-Mdm2 complex in DNA damaged cells still remain unresolved. DNA damage has been reported to cause p53 stabilization and nuclear disruption^38^, while depletion of nucleolin revealed a defect in chromosome congression^39^. In addition, several studies demonstrated the biological functions of nucleolin upon DNA damage and/or rDNA processing. However, the effects of nucleolin on those processes are still not elucidated. Therefore, we postulated that the function of HAUSP-p53-Mdm2 complex could lead to the regulation of biological functions for nucleolin to response DNA damage. The activation of the ATM-dependent DNA damage response by IR was demonstrated to regulate RNA polymerase I (Pol I) in mouse embryonic fibroblasts (MEFs)^34^. Accordingly, only DSBs caused by IR led to inhibition of Pol I transcription and displacement of Pol I from ribosomal DNA (rDNA)^34^. Interestingly, overexpression of nucleolin can repress only Pol I, but not Pol II or Pol III^35^. In the present study, we demonstrated a role for nucleolin as a new HAUSP binding protein in the DNA damage response. The formation of DSBs by IR results in a significant interaction between nucleolin and HAUSP and stabilization of nucleolin by HAUSP (Fig. 5). The increase in the HAUSP-nucleolin interaction after IR-induced DNA damage indicates that this phenomenon is possibly related to DSBs arising in the DNA damage response. Depletion of HAUSP causes an accumulation of ubiquitinated nucleolin, which is enhanced during the IR response (Fig. 5). These observations suggest that nucleolin may act as a mediator of the DNA damage response through its interaction with HAUSP. In addition to these mechanisms, a role for HAUSP was shown in several DNA damage conditions, such as UV or oxidative stress^40^,^41^,^42^. Interestingly, Schwertman and colleagues found a UV-sensitive syndrome protein UVSSA, which interacts with transcription complexes such as RNA Pol II to regulate transcription-coupled nucleotide-excision repair (TC-NER); this protein is also stabilized by HAUSP^41^. These findings suggest that HAUSP has distinct functions in multiple DNA damage response pathways.

The GST pull-down assay revealed that nucleolin associates with not only HAUSP-Mdm2 but also with HAUSP-p53. In addition, Mdm2 acts as a critical mediator between HAUSP and nucleolin (Fig. 2c). Moreover, p53 also could not interact with HAUSP and nucleolin in Mdm2-depleted cells (Fig. 2c,d). In contrary, knockdown of p53 did not affect HAUSP-nucleolin and HAUSP-Mdm2 interaction (Fig. 2e,f). A previous study suggested that Mdm2 mediates p53 stabilization through HAUSP expression, and Mdm2 and HAUSP can interact in a p53-independent manner^17^. Therefore, we considered that the blockage of self-ubiquitination of Mdm2 by nucleolin^23^ can be explained in terms of the HAUSP and nucleolin interaction. First, we hypothesized that, in the normal state, nucleolin recruits Mdm2 to HAUSP in order to stabilize Mdm2. However, the inhibition of Mdm2 expression by siRNA showed that HAUSP and nucleolin binding is reduced. This observation indicates that Mdm2 may recruit nucleolin to HAUSP, and that HAUSP then increases the stability of both Mdm2 and nucleolin via its deubiquitinating enzyme activity. We also observed a HAUSP-nucleolin interaction in Mdm2-depleted cells under IR treatment. Under a DNA damaged condition, the HAUSP and nucleolin interaction was significantly reduced by the depletion of Mdm2 (Fig. 5g,h). However, knockdown of Mdm2 did not affect the ubiquitination of nucleolin in the DNA damaged condition (Fig. 5j). These results indicate that the interaction of HAUSP-nucleolin and Mdm2 could take place in the nucleoplasm in the DNA damaged condition. ARF, one of the p53-associated proteins, sequesters Mdm2 in the nucleoplasm in response to DNA damage in order to regulate ribosome biogenesis^43^,^44^, and nucleolin is also accumulated in nucleoplasm in response to IR^33^. With this line of evidence, we propose that the sequestered Mdm2 may act as a mediator between HAUSP and nucleolin in the DNA damage response.

**Figure 6:**
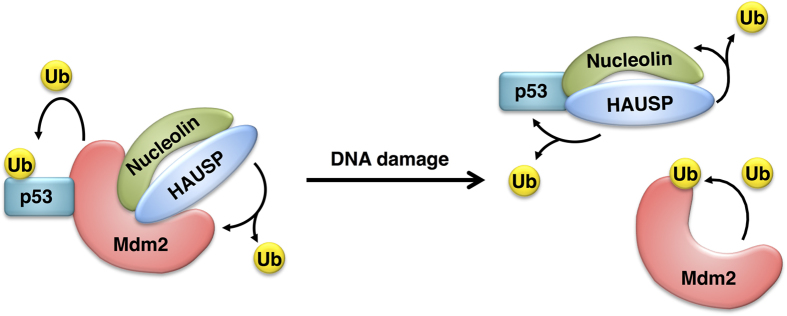
A proposed model of HAUSP-nucleolin interaction with p53 and Mdm2 upon DNA damage response. Mdm2 recruits nucleolin to inhibit self-ubiquitination and it leads to the degradation of p53 under the non-stressed condition. In the DNA damaged condition, stabilized p53 by HAUSP associates with nucleolin, increasing the stability of nucleolin by deubiquitination. Orchestration of HAUSP-p53-Mdm2 and nucleolin in cells is involved in the regulation of cell proliferation in the normal condition or DNA damage response.

We also removed p53 in the DNA damaged condition and checked HAUSP-nucleolin binding affinity. Interestingly, depletion of p53 affected the HAUSP and nucleolin interaction under the cellular stress condition but not in the normal state (Figs 2,5). Depletion of nucleolin did not affect the interaction between HAUSP and p53 in both normal and DNA damaged conditions (Supplementary Figure S3). Therefore, we suggest that p53 may predominantly regulate the HAUSP-nucleolin interaction in the DNA damaged condition. A previous study supports this suggestion as nucleolin could not be re-localized by p53 depletion in IR-treated cells^33^. Therefore, the mobility of nucleolin for its interaction with HAUSP will be important in understanding the function of p53 in cell stress responses. The HAUSP-p53-Mdm2 complex and p53-nucleolin interplay in DNA damage have been identified^18^,^23^,^45^; however, the molecular relation between these two findings was poorly understood. In this study, we showed the possibility that HAUSP plays as a linkage protein in p53, Mdm2 and nucleolin interplay upon DNA damage (Fig. 6). And our results provide a line of evidence that nucleolin may regulate the DSB response pathway as a component of a HAUSP-Mdm2-p53 complex. Obviously, HAUSP and nucleolin regulate cell proliferation in the presence of p53 in cells^9^,^23^. However, double-knockdown of HAUSP and nucleolin still affected the inhibition of cell proliferation in the absence of p53 (Fig. 4h). Although HAUSP and nucleolin are influenced by the presence of p53 to regulate cell proliferation, they might be important components for the regulation of cell proliferation. Recent studies suggest that DUBs are key regulators in DNA damage response and they can be good targets to develop therapeutic drugs for genomic diseases including cancers^46^. Our findings also suggest new insight into the molecular mechanism mediated by HAUSP as a deubiquitinating enzyme for cancer therapy in DNA damage response.

## Materials and Methods

### Cell culture, proliferation assay and ionizing irradiation

HeLa, MCF7, U2OS, HCT116 and HEK 293T cells were grown in Dulbecco’s modified Eagle’s medium (DMEM, Gibco, Grand Island, NY, USA) supplemented with 10% FBS (Gibco, Grand Island, NY, USA), 1% penicillin/streptomycin (P/S, Gibco, Grand Island, NY, USA). p53^−/−^ HCT116 cells are kindly provided by Dr. Albert J Fornace (Georgetown University). Cell proliferation ratio was measured using Cell Counting Kit-8 (Dojindo, Tokyo, Japan) according to the manufacturer’s protocol. For **γ**-irradiation, cells were plated in 6-well dish. After γ-irradiation, cells were replaced in the 37 °C incubator until the indicated times.

### Expression constructs, transfection, and antibodies

A full-length cDNAs for human HAUSP, Mdm2, annexin-1, PKM2, PP2A, and p53 were PCR-amplified from HeLa and HCT116 and subcloned into the FLAG, Myc and GST epitope encoded vectors, respectively. The HAUSP (C223S) mutant was generated by site-directed mutagenesis. FLAG-tagged nucleolin constructs were kindly provided by Dr. Borowiec (New York University School of Medicine, USA). The HA-tagged ubiquitin, ubiquitin-K48 and ubiquitin-K63 constructs have been described^47^. DNA constructs were transfected into cells by Lipofectamine 2000 (Invitrogen, Paisley, UK) according to the manufacturer’s instructions. Anti-Myc (1:200, 9E10 hybridoma cell media), anti-HA (1:200, 12CA5 hybridoma cell media), anti-FLAG (Sigma-Aldrich, St. Louis, MO, USA), anti-HAUSP, anti-p53, anti-nucleolin, anti-Mdm2, anti-β-actin, anti-ubiquitin (Santa Cruz Biotechnology, Santa Cruz, CA, USA), anti-γH2AX (Abcam, Cambridge, MA, USA) antibodies were used for immunoprecipitation, immunoblotting and immunofluorescence analysis.

### Immunoblotting and co-immunoprecipitation, ubiquitination and deubiquitination assays, and immunofluorescence assay

For immunoblotting and immunoprecipitation, the cell pellet was lysed in a lysis buffer (150 mM NaCl, 50 mM Tris-HCl [pH 7.4], 1 mM PMSF, 1 mM EDTA, 1 mM Na_3_VO_4_, 10 mM NaF, 1% Triton X-100 and protease inhibitor cocktail (PIC) tablet (Roche, Mannheim, Germany) in PBS). The resuspended cells were incubated for 20 min on ice and centrifuged at 13,000 rpm for 15 min. For co-immunoprecipitation assay, whole cell lysates were incubated with indicated antibodies for 4 hrs at 4 °C. Then protein A/G PLUS agarose bead (Santa Cruz Biotechnology, Santa Cruz, CA, USA) was added and rotated for 1 hr. The purified protein complex was separated by SDS–PAGE and transferred to a PVDF (Millipore, Bedford, MA, USA) membrane for immunoblotting analysis. For ubiquitination and deubiquitination assays, the cells were lysed in a lysis buffer (200 mM NaCl, 50 mM Tris-HCl [pH 7.4], 0.1% SDS, 1 mM PMSF, 1 mM EDTA, 1 mM Na_3_VO_4_, 10 mM NaF, 1% Triton X-100 and PIC). Cell lysates were incubated with the corresponding antibody (0.5 μg) at 4 °C overnight and then incubated with 25 μl of protein A/G PLUS agarose bead (Santa Cruz Biotechnology, Santa Cruz, CA, USA) at 4 °C for 2 hrs. The samples were washed three times with a lysis buffer and resuspended in SDS sample buffer. Immunofluorescence analysis method was previously described^21^.

### GST fusion protein purification and pull-down assays

A GST-tagged p53 fusion plasmid for expression in *E. coli* was generated by PCR fragments encoding p53 subcloned into the pGEX-4T-1 vector (Pharmacia, Chemicals, Piscataway, NJ, USA). Cells expressing the recombinant protein were lysed in a lysis buffer (10 mM Tris-HCl [pH 8.0], 150 mM NaCl, 1 mM EDTA, 200 mM PMSF, 5 mM DTT, 1% Triton X-100, 100 μg/ml lysozyme and PIC). Purification of recombinant protein and pull-down method were previously described^47^.

### RNA interference

To efficiently knockdown, the following siRNA sequences were used: HAUSP siRNA #1; 5′-CAU GCAC AAG CAG UGC UGA AGA UAA-3′, HAUSP siRNA #2 5′-AAA GUU UCC CAC CCA AAU GAC UUU G-3′, nucleolin siRNA #1; 5′-AAG CTA TGG AGA CTA CAC CAG-3′, nucleolin siRNA #2; 5′-AAG GAA ATG GCC AAA CAG AAA-3′, Mdm2 siRNA; 5′-AAC CTG AAA TTT ATT CAC ATA-3′, and p53 siRNA; 5′-AAG GAA ATT TGC GTG TGG AGT-3′. HAUSP siRNAs were synthesized (Invitrogen, Paisley, UK), and nucleolin, Mdm2 and p53 siRNAs purchased commercially (Qiagen, Valencia, CA, USA). All siRNAs were transfected at a final concentration of 20 nM using Lipofectamine RNAiMAX (Invitrogen, Paisley, UK) according to the manufacturer’s instructions.

## Additional Information

**How to cite this article**: Lim, K.-H. *et al.* HAUSP-nucleolin interaction is regulated by p53-Mdm2 complex in response to DNA damage response. *Sci. Rep.* **5**, 12793; doi: 10.1038/srep12793 (2015).

## Supplementary Material

Supplementary Figures
