# Supplementary material for: HAUSP-nucleolin interaction is regulated by p53-Mdm2 complex in response to DNA damage response
Source: Sci Rep. 2015 Aug 4;5:12793. doi: 10.1038/srep12793 (PMC4523935; doi:10.1038/srep12793)
Supplement: Supplementary Figures [file srep12793-s1.pdf]

Supplementary information for

HAUSP-nucleolin interaction is regulated by p53-Mdm2 complex in response to DNA damage response

Key-Hwan Lim, Jang-Joon Park, Bon-Hee Gu, Jin-Ock Kim, Sang-Gyu Park and Kwang-Hyun Baek*

*Corresponding Author:
Kwang-Hyun Baek
Department of Biomedical Science
College of Life Sciences, CHA University
335 Pangyo-Ro, 689 Sampyeong-Dong,
Bundang-Gu, Seongnam-Si
Gyeonggi-Do 463-400, Repulic of Korea

Tel: +82-31-881-7134
Fax: +82-31-881-7249
E-mail: baek@cha.ac.kr

Figure S1

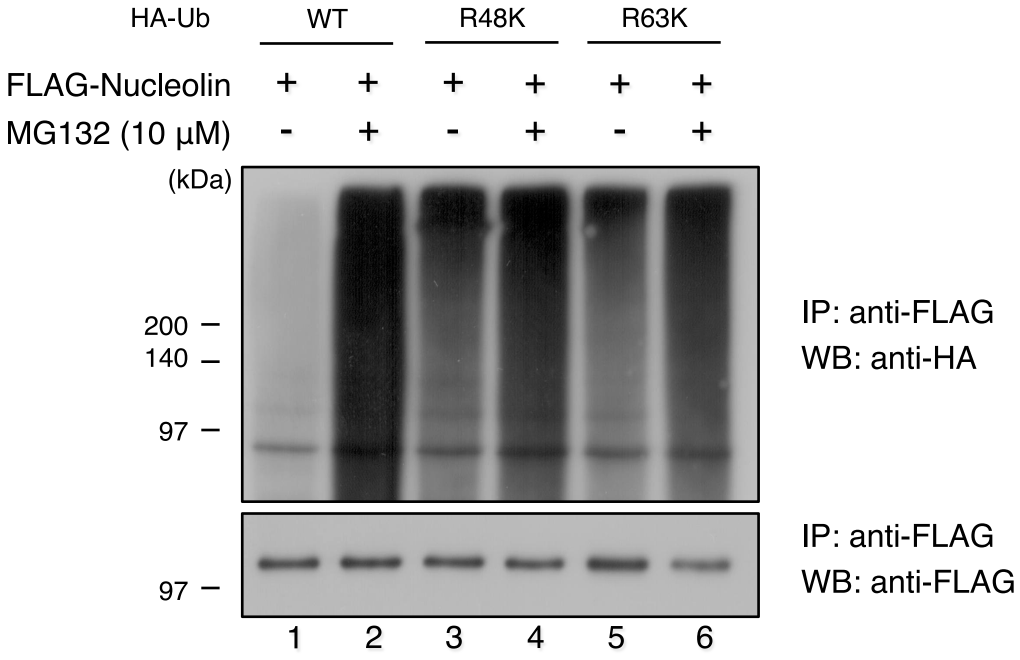
HA-Ub
WT
R48K
R63K
FLAG-Nucleolin + + + + + +
MG132 (10 μM) - + - + - +
(kDa)
200
140
97
IP: anti-FLAG
WB: anti-HA
97
IP: anti-FLAG
WB: anti-FLAG
1 2 3 4 5 6

Figure S2

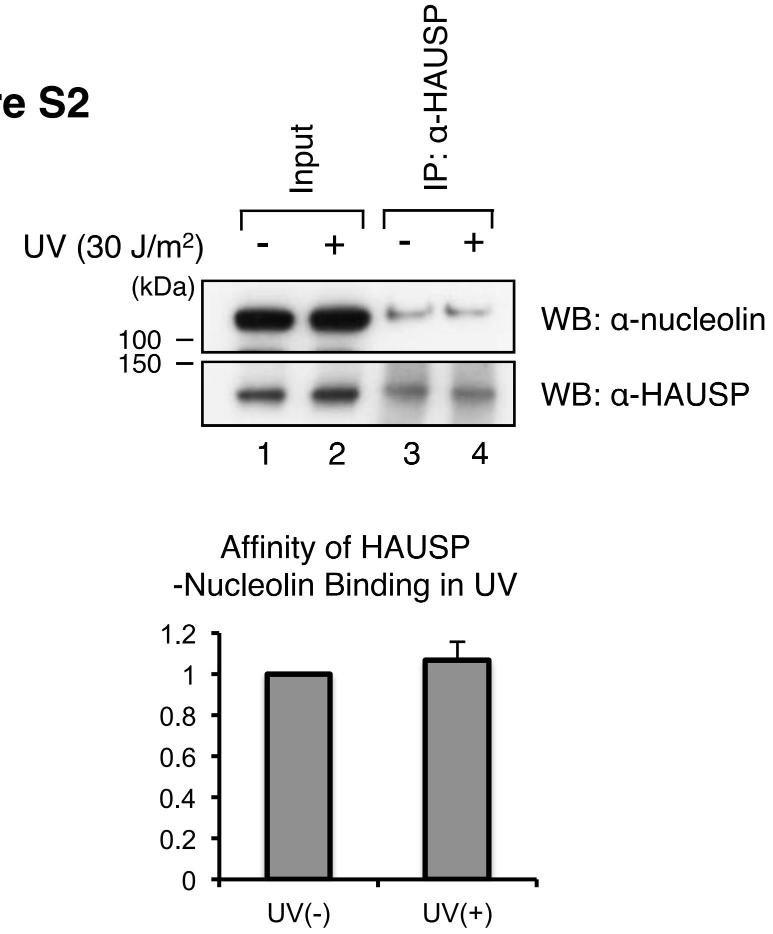
Input
IP: α-HAUSP
UV (30 J/m2)
- + - +
(kDa)
100
150
WB: α-nucleolin
WB: α-HAUSP
1 2 3 4
Affinity of HAUSP
-Nucleolin Binding in UV
1.2
1
0.8
0.6
0.4
0.2
0
UV(-)
UV(+)

Figure S3

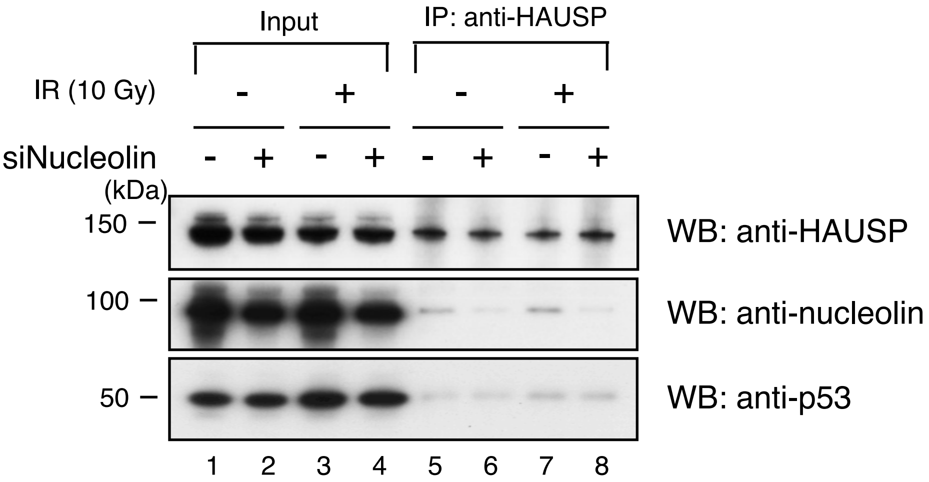

Input
IP: anti-HAUSP
IR (10 Gy) - + - +
siNucleolin - + - + - + - +
(kDa)
150
100
50
WB: anti-HAUSP
WB: anti-nucleolin
WB: anti-p53
1 2 3 4 5 6 7 8

Supplemental data figure legends

Figure S1. The Lys48- and Lys63-polyubiquitin chains of conjugated nucleolin accumulated by the 26S proteasome inhibitor MG132. HEK 293T cells expressing FLAG-tagged nucleolin and/or HA-ubiquitin, HA-ubiquitin (R48K) and HA-ubiquitin (R63K) were incubated with 10 mM MG132. Cell extracts were immunoprecipitated with an anti-FLAG antibody and subsequently immunoblotted with an anti-HA antibody.

Figure S2. The interaction of HAUSP and nucleolin in UV treatment. U2OS cell lysates exposed to UV (30 J/m^2) or controls were immunoprecipitated with an anti-HAUSP antibody. Then, HAUSP and nucleolin were detected by appropriate antibodies. Statistical data are presented as a means (n=3, *p<0.05).

Figure S3. Nucleolin does not affect the interaction between p53 and HAUSP. U2OS cells were transfected with a control and nucleolin siRNA, and then cultured for 72 hrs. Cells were treated with IR (10 Gy) during 3 hrs after transfection and harvested. Cell extracts were full-downed with an anti-HAUSP antibody, and anti-p53, anti-nucleolin and anti-HAUSP antibodies were used for immunoblotting.
